# Single-Dose Del Nido Cardioplegia *vs.* Blood Cardioplegia in Aortic Valve Replacement Surgery

**DOI:** 10.21470/1678-9741-2020-0063

**Published:** 2021

**Authors:** Haci Ali Ucak, Dilek Ucak

**Affiliations:** 1 Department of Cardiovascular Surgery, Adana City Training and Research Hospital, Adana, Turkey.; 2 Department of Anesthesiology and Reanimation, Adana City Training and Research Hospital, Adana, Turkey.

**Keywords:** Cardioplegic Solutions, Aortic Valve, Cardiopulmonary Bypass, Heart Arrest, Induced, Cardiovascular Agents, Heart Valve Prosthesis

## Abstract

**Introduction:**

In this study, we aimed to compare Del Nido cardioplegia (DNC) with blood cardioplegia (BC) in aortic valve replacement.

**Methods:**

A two-year single-institute retrospective cohort study was accomplished. Subjects who underwent aortic valve replacement surgery were divided into two groups (DNC and BC) and outcomes were compared.

**Results:**

Preoperative demographics and clinical data of the patients in both groups were similar. The time until cardiac arrest following administration of the first dose of cardioplegia was statistically significantly shorter in the BC group (47.0 sec. 25-103) than in the DNC group (63.0 sec. 48-140) (*P*=0.012). Cross-clamping time was longer in the BC group (48.7±12.3 min. *vs*. 41.5±11.8 min.) (*P*=0.041). Cardiopulmonary bypass time was statistically significantly shorter in the DNC group (BC 60.8±18.5 min., DNC 53.7±15.2 min.) (*P*=0.046). The rate of postoperative use of intravenous positive inotropic support drugs (dopamine, dobutamine, norepinephrine, etc.) for more than two hours was significantly higher in the BC group (20 [23.5%] in the BC group and nine [17.3%] in the DNC group) (*P*=0.035). Creatine kinase myocardial band and troponin I levels were slightly lower in patients receiving DNC, but no statistically significant difference was detected.

**Conclusion:**

Del Nido cardioplegia is safe and can be used efficiently as an alternative to blood cardioplegia in isolated aortic valve replacement surgery.

**Table:** 

Abbreviations, acronyms & symbols				
**AKIN**	**= Acute Kidney Injury Network **		**LV-ESD**	**= Left ventricular end-systolic diameter**
**AV**	**= Atrioventricular**		**MACCE**	**= Major adverse cardiac and cerebrovascular events**
**BC**	**= Blood cardioplegia**		**NYHA**	**= New York Heart Association**
**CK-MB**	**= Creatine kinase myocardial band**		**PRBC**	**= Packed red blood cell**
**COPD**	**= Chronic obstructive pulmonary disease**		**PW**	**= Posterior wall**
**CPB**	**= Cardiopulmonary bypass**		**RBC**	**= Red blood cell**
**DNC**	**= Del Nido cardioplegia**		**SD**	**= Standard deviation**
**EF**	**= Ejection fraction**		**STS**	**= Society of Thoracic Surgeons**
**ICU**	**= Intensive care unit**		**VF**	**= Ventricular fibrillation**
**IVS**	**= Interventricular septum**		**WBC**	**= White blood cell**
**LV-EDD**	**= Left ventricular end-diastolic diameter**			

## INTRODUCTION

Maintenance of myocardial vitality is essential in an open-heart surgery combined with cardiopulmonary bypass (CPB). Myocardial tissue is inevitably exposed to some ischemic damage during cross-clamping and surgeons aim to cause minimal damage during this ischemic process that is induced in a controlled manner. Various cardioplegia solutions exist to help myocardial tissue tolerate ischemia.

Despite containing different formulas, all cardioplegia solutions aim to minimize probable ischemia reperfusion injuries by providing cardiac depolarization and arrest with high concentrations of potassium. Blood cardioplegia (BC) is a method that has been used in clinical practice for many years, which contains 80% blood and 20% crystalloid. Del Nido cardioplegia (DNC) was first used in congenital cardiac surgery in the early 1990s^1^. It contains a base solution of a crystalloid component (Plasma-Lyte A, lidocaine, magnesium sulphate, potassium chloride, mannitol, sodium bicarbonate). Additionally, it contains 20% oxygenated autologous blood. Lidocaine is the main distinctive component of this formula. It prolongs refractory period in myocytes and minimizes sodium and calcium passage into the cell.

Although primarily developed for use in pediatric cases, the efficiency of a single dose of cardioplegia to handle an entire surgical procedure has brought about discussions for its use in adult cases. A recent meta-analysis has emphasized that the use of DNC in adult patients is a good alternative to BC^2^. Although it is stated to be safe for adult cardiac surgery, more studies are needed on specific subgroups. Conditions requiring aortic valve replacement (aortic stenosis and aortic regurgitation) should also be subject to a separate assessment in terms of their effects on the left ventricle^3,4^. However, there is still a limited number of data associated with the use of DNC in aortic valve replacement^5,6^. The aim of this study is to contribute to the literature by comparing the results of DNC and BC delivery during aortic valve replacement in adult patients.

## METHODS

This is a two-year retrospective cohort study including cases between October 2017 and September 2019. The study was approved by the local ethics committee.

Patients who underwent an additional operation in the same session due to another pathology accompanying aortic valve disease, patients operated for active infective endocarditis, and patients who underwent surgery in emergency conditions were excluded from the study. A total of 137 patients who underwent aortic valve replacement alone were included in the study. DNC was used in 52 patients while BC was used in 85 patients. All patients’ age, gender, body mass index, New York Heart Association functional status, smoking and alcohol consumption status, and preoperative comorbidity (hypertension, diabetes mellitus, dyslipidaemia, kidney failure, chronic obstructive pulmonary disease) were recorded. Preoperative echocardiographic measurements were also included in the data set. Patients with a previous history of cardiac surgery and patients who underwent combined valve, ascending aorta, or coronary surgery due to accompanying pathology were excluded from the study.

All patients underwent elective CPB under general anaesthesia. Aortic valve replacement was performed with full median sternotomy as a standard procedure. Both cardioplegia solutions were prepared according to an established protocol for each case. DNC was prepared with 13 mL lidocaine 1%, 13 mL KCl 2 mEq/mL, 13 mL NaHCO_3_ 8.4%, 1000 mL of Plasma-Lyte A, 4 mL of MgSO4 50%, and 16.3 mL of mannitol 20%. It was mixed with the blood obtained from patient in a 4/1 ratio and cooled to 4 ˚C. A dose of 20 mL/kg, with a maximum of 1000 mL, was administered through antegrade cardioplegia cannula or ostial cannula. BC solution was prepared by mixing autologous blood and crystalloid obtained from patient in a 4/1 ratio. It contained 435 mL of ringer lactate (as crystalloid), 20 mL of mannitol 15%, 20 mL of NaHCO_3_ 8.4%, and 25 mL of KCl 2 mEq/mL, equalling up to 500 mL of crystalloid in total. Each cardioplegia dose was mixed with autologous blood according to the total dose volume and the final BC solution was prepared at 24-28 ˚C. The first delivery was administered at a dose of 15 mL/kg while the subsequent deliveries were administered antegradely at a dose of 5-10 mL/kg every 15-20 minutes.

Intraoperative data and postoperative results of the patients were scanned to analyse the effectiveness of the myocardial protection strategy. Intraoperative cross-clamping and CPB times, use of inotropic support, use of intra-aortic balloon pump, electrical cardiac activity under aortic clamping, restoration of sinus rhythm, and defibrillation procedures were recorded. Troponin I and creatine kinase myocardial band (CK-MB) levels were analysed at postoperative hours 8, 24, and 48. Hourly urine output was monitored during the intensive care unit (ICU) stay. Haemoglobin, haematocrit, creatinine, creatinine clearance, and white blood cell results were recorded. Ejection fraction was measured again during routine postoperative transthoracic echocardiography. Myocardial infarction, acute kidney damage, respiratory failure, temporary or permanent neurological attacks, newly developing complete atrioventricular (AV) block, need for a permanent pacemaker, development of atrial fibrillation, reoperation due to excessive bleeding, infection development, ventilation time, and total length of ICU and hospital stay were evaluated.

## RESULTS

The present study included a total of 137 patients undergoing aortic valve replacement who met the inclusion criteria within the study period. While BC was used in 85 of these patients, DNC was used in 52. Preoperative demographics and clinical data of the patients in both groups are presented in Table 1.

**Table 1:** Patients' preoperative data.

Characteristic	BC group, n 85	DNC group, n 52
Male	51 (60 %)	34 (65.3%)
Age, years±SD	63.24±10.48	64.02±11.76
Body mass index	27.7±4.1	27.2±4.7
Preoperative comorbidities		
Diabetes mellitus	16 (18.8 %)	10 (19.2%)
Hypertension	35 (41.1 %)	19 (36.5 %)
Hyperlipidemia	13 (15.3%)	9 (17.3%)
Renal insufficiency	1 (1.17%)	0
Dialysis	1 (1.17%)	0
Asthma/COPD	8 (9.4%)	6 (11.5%)
Atrial fibrillation	6 (7.05%)	4 (7.6%)
STS risk of mortality (%)	1.95 (0.42-21.2)	1.9 (0.51-19.8)
NYHA functional status class I	5 (5.8%)	3 (5.7%)
NYHA functional status class II	26(30.5%)	14 (26.9%)
NYHA functional status class III	48 (56.4%)	30 (57.6%)
NYHA functional status class IV	6 (7. 1%)	5 (9.6%)
Preoperative echocardiographic data		
Isolated aortic stenosis	45 (52.9%)	35 (67.3%)
Isolated aortic insufficiency	7 (8.2%)	3 (5.7%)
Combined aortic valve disease	33 (38.8%)	14 (26.9%)
Ejection fraction (%)	58.8±9.8	55.4±7.9
IVS (mm)	13.8±2.6	13.1±2.4
PW thickness (mm)	12.7±1.8	12.9±1.6
LV-ESD (mm)	33.2±6.9	33.8±7.1
LV-EDD, (mm)	50.5±8.2	51.3±7.4
Preoperative blood laboratory data		
Hemoglobin (g/dL)	14.42±1.2	14.8±1.3
Hematocrit (%)	42.06±3.95	41.34±4.36
WBC counts (10^9^/L)	9.2±3.1	9.6±1.4
RBC counts (10^12^/L)	4.7±0.7	4.6±0.7
Platelet count (10^9^/L)	210.5±45.8	208.2±50.3

BC=blood cardioplegia; COPD=chronic obstructive pulmonary disease; DNC=Del Nido cardioplegia; IVS=interventricular septum; LV-EDD=left ventricular end-diastolic diameter; LV-ESD=left ventricular end-systolic diameter; NYHA=New York Heart Association; PW=posterior wall; RBC=red blood cell; SD=standard deviation; STS=Society of Thoracic Surgeons; WBC=white blood cell

Mechanical and biological valve preferences were similar in both groups. During the first dose of cardioplegia, aortic infusion method was used in 45 (52.9%) patients in the BC group and 35 (67.3%) patients in the DNC group (*P*=0.041). Ostial cannula was used in 40 (47.1%) patients in the BC group and 17 (32.7%) patients in the DNC group (*P*=0.025). There was a statistically significant difference in terms of the first route of delivery of cardioplegia in both groups. The time until cardiac arrest following administration of the first dose of cardioplegia was statistically significantly shorter in the BC group (47.0 sec. 25-103) compared to the DNC group (63.0 sec. 48-140) (*P*=0.012). The total cardioplegia volume was found higher in the BC group (1310±85.3 mL) than in the DNC group (910±105.6 mL), but there was no statistically significant difference. While all operations were completed with a single dose of cardioplegia in the DNC group, all patients in the BC group required additional doses. In the BC group, two doses of cardioplegia were used in six (7.1%) patients, three doses in 71 (83.5%) patients, and > 3 doses in eight (9.4%) patients. Cross-clamping time was longer in the BC group (48.7±12.3 min *vs*. 41.5±11.8 min.; *P*=0.041). CPB time was statistically significantly shorter in the DNC group (BC 60.8±18.5 min., DNC 53.7±15.2 min.; *P*=0.046). During cross-clamping, ventricular fibrillation was observed in eight (9.4%) patients in the BC group and three (5.7%) patients in the DNC group; *P*=0.016. The reversal time to regular sinus rhythm after the removal of the cross-clamping was statistically significantly shorter in the BC group (52.7 sec. (41-152)) compared to the DNC group (83.0 sec. (48-182)) (*P*=0.032). Additionally, the number of patients undergoing defibrillation following reperfusion was similar in both cardioplegia groups. Intraoperative data of the patients in both groups are presented in Table 2.

**Table 2:** Intraoperative data.

	BC group, n=85	DNC group, n=52	*P*-value
Mechanical valve implantation	75 (88.2%)	45 (86.5%)	0.719
Bioprosthetic implantation	10 (12.8%)	7 (13.5%)	0.720
Aortic infusion (for first dose)	45 (52.9%)	35 (67.3%)	0.041
Ostial infusion (for first dose)	40 (47.1%)	17 (32.7%)	0.025
Time to arrest (s)	47.0 (25-103)	63 (48-140)	0.012
Total cardioplegia volume (mL)	1310±85.3	910±105.6	0.013
1 dose of cardioplegia	0	52 (100%)	0.0001
2 doses of cardioplegia	6 (7.1%)	0	0.0001
3 doses of cardioplegia	71 (83.5%)	0	0.0001
>3 doses of cardioplegia	8 (8.4 %)	0	0.0001
Cross-clamping time, min	48.7±12.3	41.5±11.8	0.041
Cardiopulmonary bypass time, min	60.8±18.5	53.7±15.2	0.046
VF in cross-clamping period	8 (9.4%)	3 (5.7%)	0.016
Time to restoration of the sinus rhythm, s	52.7 (41-152)	83.0 (48-182)	0.032
No defibrillation	12(14.1%)	11(21.2%)	0.012
1 defibrillation for VF	62 (72.9%)	34 (65.4%)	0.062
2 defibrillations for VF	11(12.9%)	7 (13.5%)	0.817
> 2 defibrillations for VF	4 (4.7%)	2 (3.8%)	0.752

BC=blood cardioplegia; DNC=Del Nido cardioplegia; VF=ventricular fibrillation

The rate of postoperative use of intravenous positive inotropic support drugs (dopamine, dobutamine, norepinephrine, etc.) for more than two hours was significantly higher in the BC group (20 (23.5%) in the BC group and nine (17.3%) in the DNC group; *P*=0.035). The comparison of patients in the BC group with those in the DNC group in terms of packed red blood cell (PRBC) use revealed that blood transfusion was less common in the BC group, but there was no statistically significant difference. In the BC and DNC groups, the rate of one unit of PRBC transfusion was 10.6% and 11.5% (*P*=0.931), of two units of PRBC transfusion was 10.6% and 13.4% (*P*=0.062), whereas of > 2 units of PRBC transfusion was 5.8% and 7.7% (*P*=0.612), respectively. The total rate of chest re-exploration associated with drainage and haemorrhage was similar in both groups (2.3% *vs*. 3.8%; *P*=0.784). The comparison of complete AV block (permanent) development, postoperative left ventricular ejection fraction values, rate of new onset atrial fibrillation, ICU length of stay (hours) and hospital length of stay (days), postoperative kidney injury, major adverse cardiac and cerebrovascular events rate, myocardial infarction, and stroke incidence did not exhibit a statistically significant difference between the patients in the BC and DNC groups. The postoperative results of all patients are presented in Table 3.

**Table 3:** Postoperative outcomes.

	BC group, n=85	DNC group, n=52	*P*-value
Inotropic support > 2 hours	20 (23.5%)	9 (17.3%)	0.035
Postoperative packed red blood cells transfusion			
1 unit	9 (10.6%)	6 (11.5 %)	0.931
2 units	9 (10.6 %)	7 (13.4%)	0.062
> 2 units	5 (5.8 %)	4 (7.7%)	0.612
Total drainage, mL	680.0 (200.0-850.0)	650.0 (240.0-820.5)	0.063
Chest revision for bleeding	2 (2.3%)	2 (3.8%)	0.784
Complete AV block (permanently)	2 (2.3%)	1 (1.9%)	0.991
Postoperative EF, %	55.42±4.9	56.7±5.3	0.508
New onset atrial fibrillation	18 (24%)	21 (28%)	0.710
ICU length of stay (hours)	28.4±6.6	29.6±5.1	0.685
Hospital length of stay (days)	6.2±2.4	6.3±2.8	0.795
Renal functions			
AKIN stage 1 (creatinine increase > 0,3 mg/dL or 150%-200%)	14 (16.5%)	11 (21.2%)	0.358
AKIN stage 2 (creatinine increase > 200%-300%)	3 (3.5%)	3 (5.7%)	0.712
AKIN stage 3 (creatinine increase > 300%)	1(1.2 %)	0	0.402
MACCE	1 (1.2%)	1 (1.9%)	0.768
Mortality (30 days)	1 (1.2%)	0	0.512
Myocardial infarction	0	0	-
Stroke	0	0	-

AKIN=Acute Kidney Injury Network; AV=atrioventricular; BC=blood cardioplegia; DNC=Del Nido cardioplegia; EF=ejection fraction; ICU=intensive care unit; MACCE=major adverse cardiac and cerebrovascular events

**Fig. 1:**
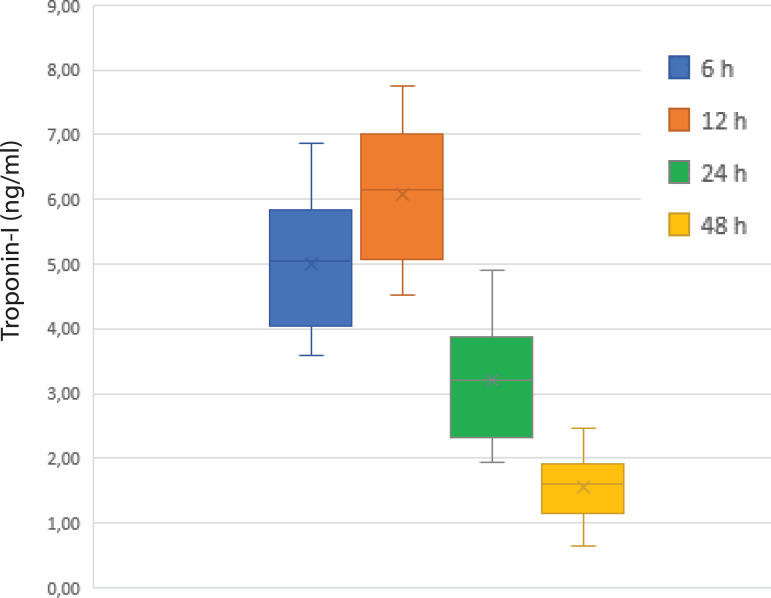
Cardiac enzyme values performed to evaluate postoperative myocardial damage in Del Nido cardioplegia group.

**Fig. 2:**
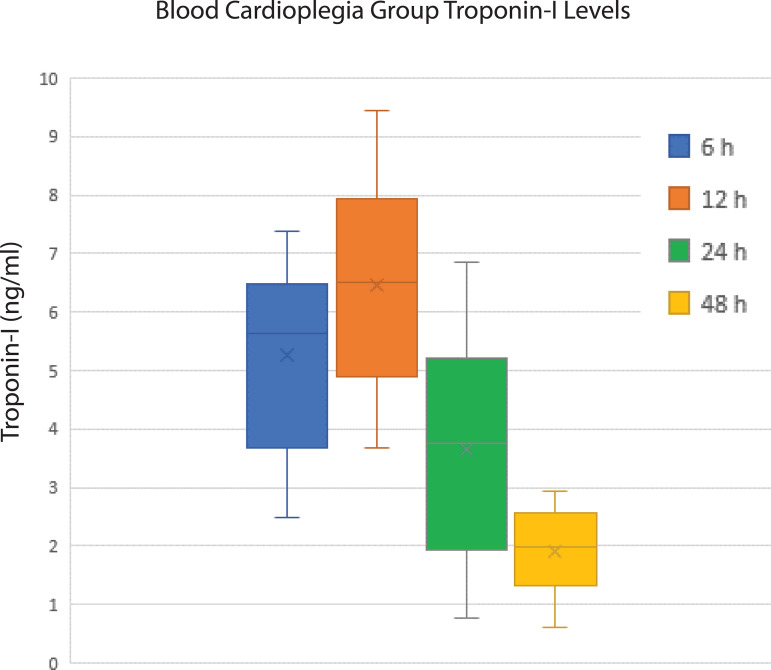
Cardiac enzyme values performed to evaluate postoperative myocardial damage in blood cardioplegia group.

The evaluation of the CK-MB and troponin I levels of the patients at six-, 12-, 24-, and 48-hour intervals revealed slightly lower values in patients receiving DNC, but no statistically significant difference was detected at any time period (Figures 1 and 2). Postoperative follow-up of cardiac enzymes in both groups are summarized in Table 4.

**Table 4:** Cardiac enzymes.

Timing	CK-MB (ng/mL)			Troponin I (ng/mL)		
	BC	DNC	*P*-value	BC	DNC	*P*-value
6 hours	26.52±9.7	22.75±7.1	0.167	5.28±2.71	4.98±3.02	0.294
12 hours	36.84±13.9	32.94±15.6	0.641	6.45±3.21	6.05±4.12	0.384
24 hours	18.08±8.5	18.23±8	0.351	3.58±2.84	3.12±3.27	0.184
48 hours	12.52±8.2	12.14±2	0.241	1.86±1.1	1.54±1.65	0.451

BC=blood cardioplegia; CK-MB=creatine kinase myocardial band; DNC=Del Nido cardioplegia

## DISCUSSION

The results of our study showed that cross-clamping and CPB times were shorter in the DNC group than in the BC group. Additionally, inotropic drug use was less frequent in the DNC group. Cardioplegic solutions used in myocardial protection strategies are mainly classified as crystalloid and BC. DNC, which can be classified as crystalloid cardioplegia despite the autologous blood component, was first used in pediatric patient groups and attracted a lot of attention upon reducing the need for repeated doses^7^. Medical professionals also began to use DNC in this field after several studies in which DNC was compared with classical cardioplegia methods in adult patient groups and revealed no negative clinical effect^2,8^. It has become increasingly popular as a single dose of cardioplegia enables to carry through many cardiac surgery procedures in adult patients and allows the surgeon to maintain high concentration during the procedure. We previously published the results of a study presenting our experience in coronary surgery with DNC, which we have been using in adult patients in our clinic since 2014^9^. In the present study, we compared the DNC method with classical BC in patients undergoing isolated aortic valve replacement and evaluated its safety and efficacy.

In our study, no additional dose of cardioplegia was needed in any of the operations with DNC, which allowed the operations to continue without any interruption in the DNC group, resulting in shorter cross-clamping and CPB times; thus, we achieved consistent results with our hypothesis. The difference between the two groups regarding the use of ostial cannula was solely due to the higher number of patients with aortic insufficiency in the DNC group. Ostial cannulation was performed for all patients with aortic insufficiency. In the BC group, all doses following aortotomy were administered through coronary ostia even if the first dose was administered through aortic root. No patient had a major traumatic injury affecting the course of surgery in either group. Although theorized to cause tolerable damage, there has been no satisfactory study investigating the long-term effects of coronary perfusion cannulas on intima. In this aspect, outright delivery of cardioplegia in a single penetration will at least reduce the risk of trauma.

In our study, electrical activity terminated sooner in the BC group following the administration of cardioplegia. Sanetra et al.^10^ carried out a study comparing cold BC and DNC, and their results were contradictory to ours. The same cardioplegia formulas were used in both studies, but Sanetra et al.^10^ applied BC at +4 ˚C while we applied it at 24-28 ˚C. Such difference may have reduced the membrane permeability and resulted in prolonged attainment of effective intracellular values for the BC solution, which has a higher content of formed blood elements^11^. Diastolic arrest and depolarization inevitably increase the amount of intracellular sodium and calcium, resulting in myocardial cell damage^12^. It appears that the use of autologous blood as cardioplegia is the most plausible option since it is more physiological and has high oxygen-carrying capacity and lower haemodilution, contrary to its crystalloid counterparts. However, no clear superiority has been demonstrated in any comparative studies with BC and crystalloid-based formulas^13,14^. A current meta-analysis has indicated that smaller volumes of cardioplegia should be used in patients receiving DNC^8^. Consistently, the volume of cardioplegia was lower in the DNC group. Some studies reported that smaller volume of PRBC transfusion was associated with decreased haemodilution due to lower cardioplegia volumes^15,16^. Our data did not only coincide with these results, but also lower PRBC use was observed in the BC group, but this difference was not statistically significant. It should not be overlooked that DNC contains 4/1 autologous blood. Haemodilution is indeed an important parameter, however, there are many factors affecting PRBC transfusion and more comprehensive studies are needed in this regard^17^.

Maintenance of stable cardiac arrest during surgery is also an important part of myocardial protection strategies. DNC offers a different form of myocardial protection with its class 1b anti-arrhythmic agent lidocaine; lidocaine blocks sodium channels, thus, it prolongs the refractory period and reduces the negative effects of myocardial arrest by decreasing intracellular sodium and calcium accumulation^18^. Besides, the crystalloid fluid in the DNC formula does not contain calcium; its 20% blood component is the only source of calcium administered to patients. Not only that, but also magnesium sulphate contributes to the reduction of intracellular calcium by competitively blocking the passage of calcium through the cell membrane^19^. In an animal experiment, Govindapillai et al. showed that the resting membrane potential exhibits higher voltage and is less responsive to spontaneous and inducible stimulations during the ischemic period^20^. As a matter of fact, ventricular fibrillation was also observed less frequently during the cross-clamping period in the DNC group. In our study, no significant difference was observed between the two groups in CK-MB or troponin I levels at any period. Although previous studies reported the positive effects of DNC in increasing troponin I levels, most of them included operations requiring myocardial resection (such as mitral valve replacement), which makes it difficult to evaluate the results homogenously^21-23^. A recent meta-analysis has revealed that spontaneous defibrillation is associated with the use of DNC^24^. This may also explain the lower release of troponin I associated with less defibrillator use.

### Limitations

Our study is a retrospective cohort study with a single-centre, nonrandomized, limited number of patient groups. In this respect, it does not include long-term patient follow-up and long-term echocardiographic follow-up for myocardial protection. We observed small differences that were not statistically significant in terms of length of ICU and hospital stay, postoperative myocardial infarction, stroke, and renal functions, however, larger patient series are needed to obtain a better understanding in this regard. We didn’t perform a cost analysis and future studies are needed in this field. Our study is unable to reveal any new findings with respect to optimal timing for a second dose or the reliability of the second dose since all participating patients in the DNC group were operated with a single dose of cardioplegia. Studies on more complex cases with longer cross-clamping times are needed to evaluate the results of DNC use, which is gaining more popularity every day. In the future, objective data on myocardial perfusion in an outright delivery of cardioplegic solution will also help to elucidate unanswered questions.

## CONCLUSION

Ultimately, all these evaluations along with the comparison of clinical results indicate that DNC is an equivalent alternative to BC in isolated posterior valve replacement operations in adult patient groups.

**Table:** 

Authors' roles & responsibilities	
HAU	Substantial contributions to the conception or design of the work; or the acquisition, analysis or interpretation of data for the work, drafting the work or revising it critically for important intellectual content; final approval of the version to be published
DU	Substantial contributions to the conception or design of the work; or the acquisition, analysis or interpretation of data for the work; final approval of the version to be published
